# Succession of Intestinal Microbial Structure of Giant Pandas (*Ailuropoda melanoleuca*) during Different Developmental Stages and Its Correlation with Cellulase Activity

**DOI:** 10.3390/ani11082358

**Published:** 2021-08-10

**Authors:** Aishan Wang, Mingye Zhan, Enle Pei

**Affiliations:** 1 Shanghai Zoological Park, Shanghai 200335, China; zoowas@126.com; 2 Institute of Pollution Control and Ecological Safety, College of Environmental Science and Engineering, Tongji University, Shanghai 200092, China; 1910046@tongji.edu.cn

**Keywords:** giant pandas (*Ailuropoda melanoleuca*), development, diversity, dominant microbes, cellulase

## Abstract

**Simple Summary:**

Giant pandas (*Ailuropoda melanoleuca*) are endangered animals and are uniquely inhabitant in China. These rare animals have gradually developed bamboo-eating adaptability through persistent evolution. Intestinal microbes play an important role in the digestion, absorption, metabolism, and development of giant pandas especially by facilizing the degradation of bamboo polysaccharides such as cellulose. Currently, genes directly related to cellulose degradation have not been identified in the genome of giant panda, and cellulose digestion is therefore likely dependent on intestinal microbes. This study analyzed the changes in intestinal microbial structure of giant pandas (cubs, sub-adults, and adults) in different developmental stages. The impact was also assessed with the changes in food composition probed into the succession regularity of intestinal microbes and the activities of intestinal flora on the digestion and utilization of cellulose in bamboo.

**Abstract:**

The interaction between intestinal microbial flora and giant pandas (*Ailuropoda melanoleuca*) is indispensable for the healthy development of giant pandas. In this study, we analysed the diversity of bacteria and fungi in the intestines of six giant pandas (two pandas in each development stage) with a high-throughput sequencing technique to expand the relative variation in abundance of dominant microbes and potential cellulose-degradation genera in the intestines of the giant pandas and to explore the correlation between dominant microbial genera in the intestines and cellulose digestion activities of giant pandas. The results showed that the intestinal bacterial diversity of young giant pandas was higher than that of sub-adult and adult giant pandas, and Shannon’s diversity index was about 2.0. The intestinal bacterial diversity of giant pandas from sub-adult to adult (mature stage) stage showed an increasing trend, but the intestinal fungal diversity showed no considerable regular relations with their ages. The microbial composition and abundance of giant pandas changed in different developmental stages. Pearson correlation analysis and path analysis showed that there was a close relationship between the dominant microbes in the intestines of giant pandas, and the interaction between microbial genera might affect the cellulose digestion ability of giant pandas. Generally, the digestibility of cellulose degraders in pandas was still insufficient, with low enzymic activity and immature microbial structure. Therefore, the utilization and digestion of bamboo cellulose still might not be a main source of energy for pandas.

## 1. Introduction

Intestinal microbes are critical in nutrient absorption [1,2,3], metabolism and immunity [4,5,6]. The initial colonization of microbes is sourced from diet or through maternal–neonatal transmission [7] during the early stages of development. Giant pandas are unique endangered species in China, and it is the key objective of wildlife conservation research in the world to maintain panda colonies. As is the case in humans, intestinal microbes play critical roles in the growth and development of giant pandas [8]. Giant pandas have the intestinal characteristics of carnivorous animals with short and straight intestines without rumen structure. However, giant pandas mainly eat bamboo with high fibre contents. Since they do not have cellulose-degrading enzymes, the cellulose degradation in pandas mainly depends on intestinal microbes [9,10,11,12].

The adaption to bamboo diet and cellulose digestibility by giant pandas is closely related to the floral structure of intestinal bacteria and fungi and is partially influenced by external environments such as altitude and season [13,14]. However, Zhang et al. [15] found that the abundance of cellulase genes in intestinal microflora of giant panda is lower than other carbohydrate-metabolizing genes. Therefore, whether cellulose is the digestive and useful nutrition source for the dominant microbes of giant panda after eating bamboo is still uncertain. In addition, the differences between captive and wild environments also affect the intestinal microbial structure of giant pandas. Yao et al. [16] have found that intestinal microbiome of giant panda cubs in a captive environment was different than pandas released into the wild environment. Besides these external factors, microbial interaction may regulate the structure and diversity of gut microbes, further affecting the metabolism of the host [17,18]

Recently, several studies have been conducted to identify key roles of the dynamic changes of intestinal flora in the healthy development of giant pandas, especially in the different stages of cub development. Guo et al. [19] found that during the giant panda cubs’ (0–1 years old) development and a substantial change in food, their intestinal flora structure was evident, especially the microbial diversity increased significantly in the first half year of age. The microbial structure of pandas that have consumed bamboo after the first year of age was different from that of panda, which was still fed with formula milk [20].

However, there are few reports on the dynamic changes in intestinal flora structure in different stages (cubs, sub-adults, adults) of giant pandas and the succession regularity of intestinal microbes in the whole growth and development process of giant pandas. In addition, in the long-term bamboo-eating process, it is still questionable whether the giant panda domesticates intestinal predominant microbes through the high content of cellulose in bamboo and its correlation with cellulose digestion.

In this study, a total of six captive giant pandas at different growth stages (two cubs, two sub-adults, and two adults) with similar living environments were fed with a bamboo diet. The structure and abundance of intestinal bacteria and fungi of giant pandas at different stages of growth were analysed by high-throughput sequencing. Their cellulose digestibility, the change regularity of intestinal floral structure, and its relationship with cellulose digestibility during the growth and development of giant pandas were further studied in order to provide scientific insights into the feeding and protection of giant pandas.

## 2. Materials and Methods

### 2.1. Research Object

In this study, six captive giant pandas (cubs, sub-adults, adults) in different stages of development were kept in Shanghai Zoo and Shanghai Wild Animal Park. During the sampling period (from August 2017 to February 2018), the sampled giant pandas were all in good condition during the total of two sampling processes, and there were no abnormalities such as illness or mucus excrement, and no medication was taken during this study. Some of the key characteristics of giant pandas are listed in Table 1. In addition, panda cubs feed mainly on milk, supplemented with bamboo shoots, coarse pastry, carrot, and apple. Cubs older than 1 year old can eat 7~8 kg of bamboo per day; 4 to 5 years old sub-adult pandas can eat 30~40 kg of bamboo per day, which is nearly the amount of bamboo consumed daily in an adult, namely 40–50 kg.

### 2.2. Sample Collection and Pretreatment

Fresh stool samples of 6 captive giant pandas were collected and were numbered from S1–S6. We collected the stool in an enclosure to avoid environmental interference every morning for five consecutive days in each sampling month (August 2017 and February 2018). The stool was stored in frozen condition and unfrozen with ice bags before use. For each panda sample, a sterile spoon was used to collect 10 g/d of the pollution-free core of each stool sample, which had been collected and stored in the freezing condition during the aforementioned 5 consecutive days. The cores were fully mixed as one new stool sample of the giant panda and were used to extract the total DNA of microbes for microbial diversity analysis. We also stored every stool sample at 4 °C, and the cellulase activity was assessed.

### 2.3. Analysis of Intestinal Cellulase Activity of the Giant Pandas

A total of 5 g of giant panda stool stored in cold condition was taken into a 50 mL Erlenmeyer flask, and 5 mL of 1% carboxymethyl cellulose solution was added. Afterward, 5 mL of acetate buffer solution (pH 5.5), was added to the culture and the mixture was kept at 37 °C for 72 h before measuring the enzymatic activity at 540 nm by DNS colorimetry method. We set up parallel experiments (n = 3) and made no-substrate control group tests (using distilled water to replace the carboxymethyl cellulose solution). The glucose standard curve used was y = 0.6099 × −0.0059, *R*^2^ = 0.9935. The production of glucose released by hydrolysing a total of 1 μg faecal sample in 1 h was defined as 1 enzyme activity unit (U), and the determination conditions were modified appropriately by referring to the relevant literature of soil cellulase activity analysis [21,22]

### 2.4. Analysis of Intestinal Microbial Diversity of Giant Pandas

Frozen samples were used to extract DNA using PowerSoil^®^ DNA Isolation Kit (Mo Bio Laboratories Inc., Carlsbad, CA, USA) kit and the concentration was determined using NanodropTM 2000 Spectrophotometer (Nanodrop, Wilmington, DE, USA) before storage at −20 °C. Bacteria were amplified using 338F (5′-ACTCCTACGGGAGGCAGCAG-3′) and 806R (5′-GGACTACHVGGGTWTCTAAT-3′) primers in a master mix of 20 μL: 5 × Fastpfu Buffer 4 μL, 2.5 mM dNTPs 2 μL, FastPfu Polymerase 0.4 μL, primers each 0.8 μL, BSA 0.2 μL, template 10 ng, and supplemented with ddH_2_O. Specifically, fungi were amplified using ITS1F (5′-CTTGGTCATTTAGAGGAAGTAA-3′) and ITS2 (2043R) (5′-GCTGCGTTCTTCATCGATGC-3′) in a reaction mix of 10 × Buffer 2 μL, 2 μL of 2.5 mM dNTPs, 0.2 μL of rTaq Polymerase, 0.8 μL of each primer, 0.2 μL of BSA, and 10 ng of template, filled with ddH_2_O. Reaction thermoprofile include 95 °C for 3 min, 95 °C for 30 s, 55 °C for 30 s, 72 °C for 45 s (27 cycles for bacteria and 35 cycles for fungi), 72 °C for 10 min, 10 °C to end the reaction. The amplified products were further purified and separated using 2% agarose gel.

Purified amplicons were pooled in equimolar and paired-end sequenced (2 × 250) on an Illumina MiSeq platform was performed according to the standard protocols. The raw reads were deposited into the NCBI Sequence Read Archive (SRA) database. Raw fastq files were demultiplexed and quality-filtered using QIIME (version 1.17) with the following criteria: (i) The 300 bp reads were truncated at any site receiving an average quality score <20 over a 50 bp sliding window, discarding the truncated reads that were shorter than 50 bp. (ii) Exact barcode matching, 2 nucleotides mismatch in primer matching, reads containing ambiguous characters were removed. (iii) Only sequences that overlapped longer than 10 bp were assembled according to their overlap sequence. Reads that could not be assembled were discarded. Operational Units (OTUs) were clustered with 97% similarity cut-off using UPARSE (version 7.1, http://drive5.com/uparse/, accessed on 15 October 2017), and chimeric sequences were identified and removed using UCHIME. The taxonomy of each 16S rRNA gene sequence was analysed by RDP Classifier (http://rdp.cme.msu.edu/, accessed on 3 May 2018) against the silva 16S rRNA database and UNITE ITS database using confidence threshold of 70% [23].

### 2.5. Statistics Analysis

The microbial α-diversity in the representative data was calculated with OTU abundance, the dominant microbial taxa were calculated at the genus level, and the cellulase data were analysed by OriginPro 8.0. All data were expressed by the mean value and standard error (n = 3), with significant difference expressed as *p <* 0.05. Pearson correlation analysis and path analysis were carried out between cellulase activity and characterized intestinal bacterial diversity during the food transformation period of giant pandas by SPSS software. Correlation coefficient, direct path coefficient and indirect path coefficient were used to reflect the correlation degree between the two variables. Among them, simple correlation coefficient (r_iy_) analysed by Pearson correlation and direct path coefficient (P_iy_) were obtained by regression analysis processed by SPASS 22.0. The indirect path coefficient was calculated with the product of r_iy_ and P_iy_.

## 3. Results

### 3.1. Intestinal Microbial Diversity of Giant Pandas at Different Developmental Stages

**Figure 1:**
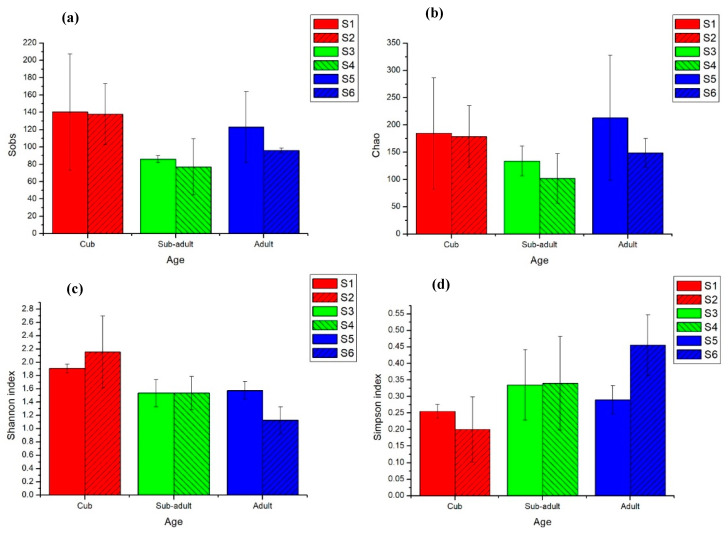
Intestinal bacterial Alpha diversity index of giant pandas at different developmental stages, taking the average of two experimental results in Aug (2017) and Feb (2018) of each sample. (**a**) Sobs; (**b**) Chao; (**c**) Shannon’s diversity index; (**d**) Simpson’s diversity index.

The library coverage was more than 99.8%, and the coverage was substantial for the microbial diversity (Table S1 and Figure 1). According to the Sobs (the number of species observed) index and Chao index of bacterial OTU level, the richness of intestinal bacterial flora changed during the development of giant pandas. This highlighted that the intestinal bacterial richness of giant panda cubs and adults was higher than that of sub-adults. Compared with sub-adults, the number of OTU in the intestines of adult giant pandas was higher (according to Sobs index and Chao index). This relationship existed among the samples collected from adult giant pandas, for the richness of bacterial flora, S5 > S6, and for the panda’s age, S5 < S6.

According to Shannon’s diversity and Simpson’s diversity indices, the intestinal bacterial diversity of giant panda cubs was higher than that of sub-adults and adults, and the average value of cub samples was approximately 2.0. After the shift from milk feeding to bamboo feeding, Shannon’s diversity index of intestinal bacteria in giant pandas of about 1 year old was as high as 2.25 (on average). On the other hand, Shannon’s diversity index of intestinal bacteria in giant pandas, which developed at 1 and a half years old was similar to that of sub-adults and adults. Interestingly, the diversity and heterogeneity of intestinal bacteria between sub-adult and adult giant pandas were similar.

**Figure 2:**
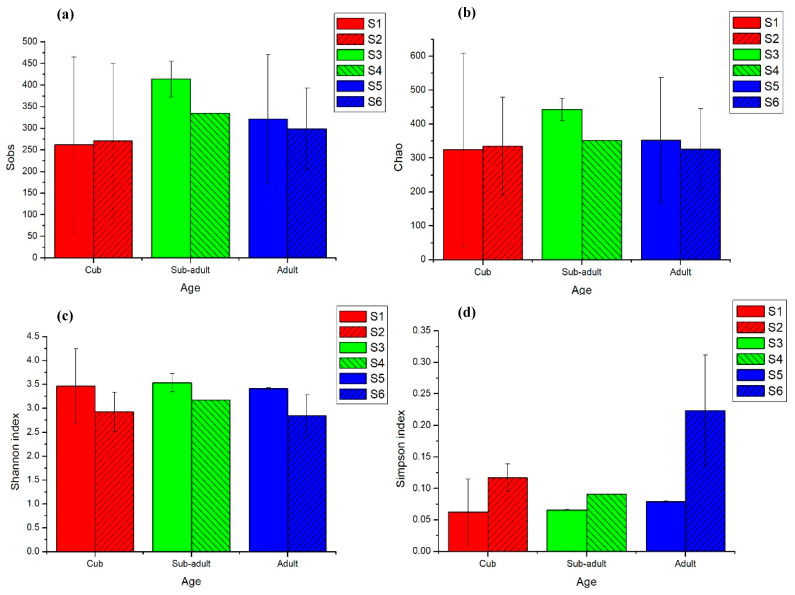
Intestinal fungal Alpha diversity index of giant pandas at different developmental stages, taking the average of two experimental results in Aug (2017) and Feb (2018) of each sample. (**a**) Sobs; (**b**) Chao; (**c**) Shannon’s diversity index; (**d**) Simpson’s diversity index.

The analysis of fungal diversity in stools of giant pandas at different developmental stages are outlined in Table S2 and Figure 2. According to Sobs and Chao indices for fungi OTU level, during the development of giant panda, the richness of fungal flora in the sub-adult stage was higher than that in the cub and adult stage. According to Shannon’s diversity and Simpson’s diversity indices, the diversity and heterogeneity of intestinal fungi in giant pandas were complex. However, no obvious regularity related to age was observed. There were certain differences between S6 (Youyou) and the other individuals, the Simpson’s diversity indices of intestinal fungi were generally below 0.15, which revealed that the intestinal dominant fungal taxa were prone to be evenly distributed in pandas without any significantly dominant fungi genera in the intestines of giant pandas except for S6.

Therefore, changes in the diversity of gut microbes were linked to the growth of giant panda, especially the bacteria. During the stage of cub and the prime of life, in which the metabolizing conditions were stronger, the diversity of gut bacteria was higher than that of other developmental stages. The fluctuation in diversity of fungi was the opposite to that of bacteria in pandas, which suggests that there might be a strict relationship between bacteria and fungi in the gut within a limited space. The dominant bacterial genera gradually occupied a large proportion, whereas the fungal genera were evenly distributed in pandas as their age increased. This indicates that specific microbial structure might be shaped with the growth of individuals and intestinal bacteria, and these more likely interact with their hosts.

### 3.2. Succession of Age-Structure-Abundance of Dominant Microbes in the Intestines of Giant Pandas

**Figure 3:**
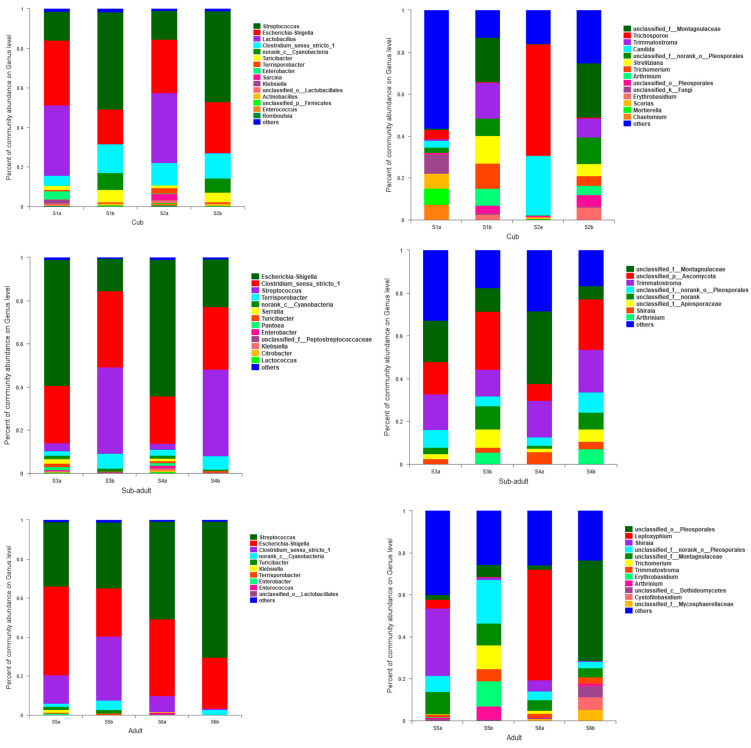
Bar diagram of intestinal dominant bacteria (**left**) and fungi (**right**) abundance in genera of different developmental stages for giant pandas.

The predominant genera of bacteria in the intestine of giant panda cubs during the panda period were *Streptococcus* (accounting for 35.2–38.1%), *Escherichia–Shigella* (22.3–26.1%), *Clostridium_sensu_stricto_1* (11.5–12.3%), *Lactobacillus* (11.3–12.2%), and *Turicibacter* (4–5%). The most dominant bacterial genera in the intestine during the sub-adult period were *Escherichia–Shigella* (37.2–41.4%), *Clostridium_sensu_stricto_1* (25.7–30.8%), *Streptococcus* (21.5–22.7%), *Terrisporobacter* (4–5%), and *Serratia* (0.5~1%). On the other hand, the dominant bacterial genera in the intestine during adulthood were *Streptococcus* (33.3–62.3%), *Escherichia–Shigella* (31~36.4%), *Clostridium_sensu_stricto_1* (3.5~22.6%), *Turicibacter* (0–1%), and *Klebsiella* (0–0.7%) (Figure 3).

Others contain some microbes whose relative abundance is less than 0.05 in samples. Both a and b represent sampling time.

The dominant intestinal fungal genera of giant panda pups were Unclassified_f_Montagnulaceae (accounting 15.7–18.3% of the total), *Trichosporon* (1.2–16.1%), *Trimmatostroma* (6.2–12.6%), *Candida* (1–8.5%), and Unclassified_f_norank_o_Pleosporales (6.8–9%). The sub-adult giant panda intestinal dominant fungal genus was Unclassified_f_Montagnulaceae (accounting for 15.7–21.1% of the total), Unclassified_p_Ascomycota (15.1–20.4%), Trimmatostroma (15.1–19.1%), Unclassified_f_norank_o_Pleosporales (6.5–6.6%), and Unclassified_f_Apios (3.5–5.2%). The dominant genera of intestinal fungi in adult giant pandas were Unclassified_o_Pleosporales (3.8–26.4%), *Leptoxyphium* (2.5–24.6%), Shiraia (2.8–19%), Unclassified_f_norank_o_Pleosporales (3.2–13.5%), and Unclassified_f_Montagnulaceae (4.8–10.5%) (Figure 3).

**Figure 4:**
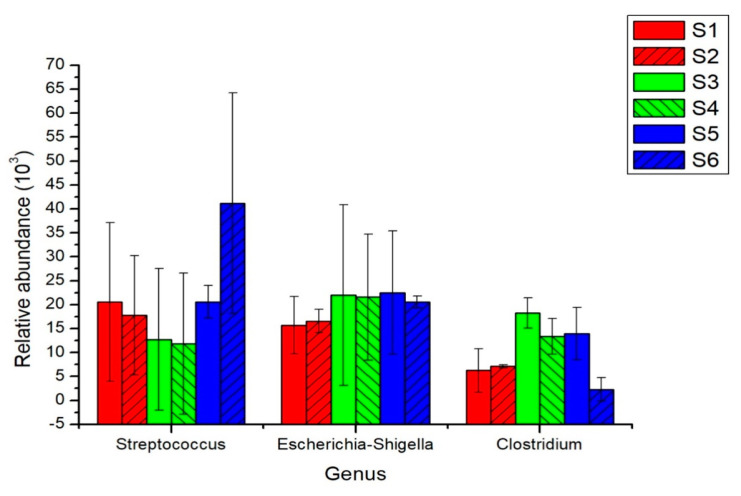
Abundance of three dominant bacteria in the gut of giant pandas at different developmental stages. Take the average of two experimental results in Aug (2017) and Feb (2018) of each sample.

It could be seen that the dominant genus of bacteria in the intestine of giant pandas was predominant, but the proportion of the dominant genus of fungi generally did not exceed 25%, which was consistent with the results of diversity. The dominant genera of intestinal bacteria of giant pandas in different developmental stages were mainly Streptococcus, Escherichia–Shigella and Clostridium_sensu_stricto_1, and there were differences in the specific percentages of different developmental stages (Table S3 and Figure 4).

A specific trend was obviously in the order of Streptococcus > Escherichia-Shigella > Clostridium_sensu_stricto_1 in the intestinal tract of young cubs. The order in the sub-adult intestinal tract was Escherichia-Shigella > Clostridium_sensu_stricto_1 > Streptococcus whereas in the adult intestine, it was Streptococcus > Escherichia-Shigella > Clostridium_sensu_stricto_1. In addition, the intestinal dominant bacterial structure was similar between different individuals at the same developmental stage. It was evident that the dominant bacterial structure in the panda’s intestine may be the result of long-term evolution of the giant panda.

For the giant panda cubs, *Lactobacillus* was also dominant in the intestines, accounting for 35.6%. This was primarily attributed to the giant panda cubs, which were fed with breast milk or other dairy products as their main food source. In addition, some small amounts of genera were also distributed by staged characteristics; for example, *Actinobacillus*, *Romboutsia* and *Sphingobacterium* prefer to colonize in cubs such as *Enterobacter,* and *Klebsiella* are dominant in both cubs and sub-adults. The *Serratia*, *Pantoea* and *Raoultella* were only found in sub-adults such as *Weissella* and *Vagococcus* were only found in adults.

*Streptococcus* showed a trend of decreasing at first and then increasing during the development of giant pandas. The amount of *Streptococcus* was rich in the sub-adult-to-adult development stage; however, it was strange that the abundance of *Streptococcus* in the cub to sub-adult stage decreased at first. It was accompanied by the increase in abundance of other bacteria such as *Clostridium_sensu_stricto_1*, *Escherichia-Shigella*, etc. Whether the interaction between bacterial genera or other external factors had the potential to affect the status of these predominant genera requires further study. The abundance of *Clostridium_sensu_stricto_1* changed with the age of giant pandas with the order of sub-adults > adults > cubs. The population quantity of *Escherichia-Shigella* was stable during the development of giant pandas, especially after the sub-adult stage.

The structure of intestinal dominant fungi showed no considerable changes with age. The proportion of dominant fungi in different development stages and different individuals varies greatly, and the fungal populations were rich and evenly distributed with low predominance, consistent with the results of α diversity of fungal community in pandas.

**Figure 5:**
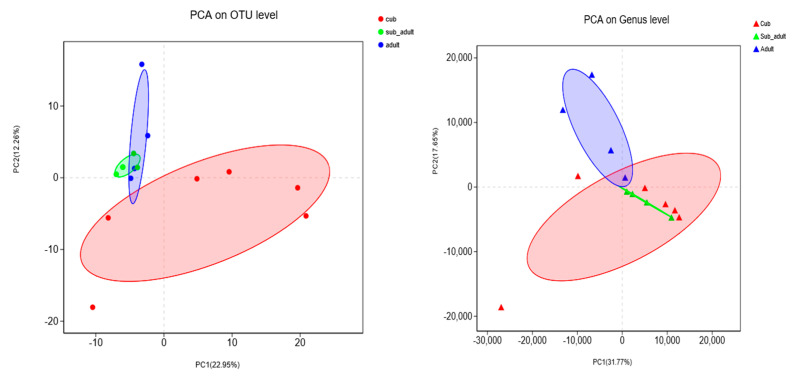
PCA Analysis of Intestinal Bacterial (**left**) and Fungal (**right**) Communities in Giant Pandas at Different Developmental Stages.

PCA analysis indicated that the flora structure of bacteria and fungi in the intestines of giant pandas in each development stages were similar (Figure 5). Interestingly, in different development stages, the similarity of bacterial flora between sub-adults and adults was higher than between cubs, and the similarity of fungal flora between cubs and sub-adults was higher than that between adults.

As shown previously, the structure of bacterial community in pandas was gradually stabilized with growth. The status of the dominant genera was also determined, and changes in their abundance had staged characteristics, whereas the fungal community in pandas was very likely influenced by environmental factors, because the twin cubs or twin sub-adults that held the same living and raising conditions, had more similar fungal components.

### 3.3. Correlation between Cellulose Digestibility and Intestinal Microbial Structure in Giant Pandas

**Figure 6:**
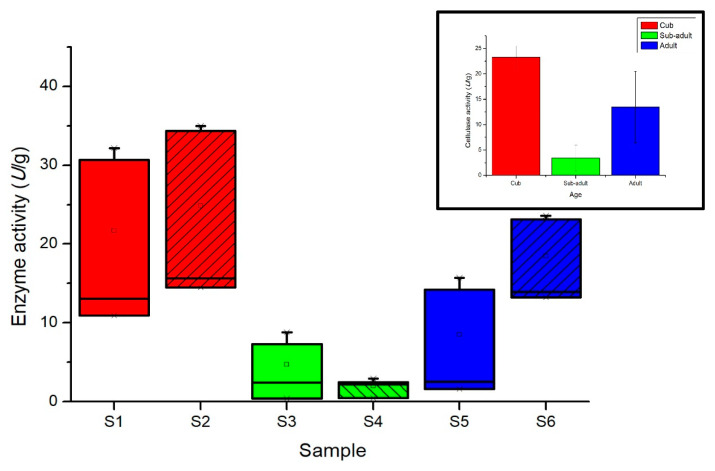
Intestine cellulase activity (the values corresponding to average ± standard deviation) of giant panda at different developmental stages. Take the average of two experimental results in Aug (2017) and Feb (2018) of each sample.

During the growth of giant pandas, the intestinal cellulase activity changes obviously in different periods (Table 2 and Figure 6); the cellulase activity of cubs was higher than other developmental stages, and sub-adults showed the lowest activity.

Combining the above-mentioned results of intestinal dominant bacterial flora structure, it was found that the abundance of the dominant bacteria genera also changed greatly during the growth from cub to sub-adult, and the abundance remained stable or increased during the development from sub-adult to adult (such as S5). In order to further understand the key role of dominant microbes in digesting cellulose and the corresponding interaction effect of intestinal microbes in giant pandas, the cellulase activity of giant pandas was correlated with the relative abundance of several intestinal dominant microbes, and then the following results were obtained by correlation analysis and path analysis (Table S4 and Table 3).

According to the results of Pearson correlation analysis, it was noticed that the dominant intestinal bacteria and fungi that were closely related to the panda cellulase activity include *Escherichia-Shigella*, *Streptococcus* and *Clostridium*_sensu_stricto_1 and Unclassified_f_Apiosporaceae. In addition, there was a close relationship between the panda intestinal microbes, including bacteria and bacteria, fungi and fungi, and bacteria and fungi. A negative correlation between Streptococcus and Escherichia-Shigella with a correlation coefficient of −0.859 (*p <* 0.05) was observed. The Unclassified_o_Pleosporales was negatively correlated with Unclassified_f_Montagnulaceae; however, it was positively correlated with Leptoxyphium (*p <* 0.05). *Streptococcus* was significantly negatively correlated with Unclassified_f_Apiosporaceae (*p <* 0.05).

Path analysis of dependent variable intestinal cellulase activity (Cellulase, y) and independent variables *Escherichia-Shigella* (x1), *Streptococcus* (x2), Unclassified_f_Apiosporaceae (x3), and *Clostridium*_sensu_stricto_1 (x4) was conducted (Table 3). The interaction between the predominant flora in the intestinal tract of the giant panda was observed. Among them, *Streptococcus, Escherichia-Shigella* and *Clostridium*_sensu_stricto_1 all changed their correlation with cellulase activity after interacting with other dominant bacteria in the intestine of giant pandas; in particular, *Streptococcus* processed a positive correlation with the panda intestinal cellulase. However, cellulose degraders bacteria in the rumen or other high-cellulose conditions such as *Flavobacterium*, *Pedobacter* and *Pseudomonas* and *Paenibacillus* [24] and *Sphingobacterium* [25] were in extremely low abundance especially in adult pandas with bamboo diet.

Therefore, during the long-term domestication of bamboo-eating pandas, the structure of functional microbial communtiy became complex and remained immature. Although the dominant bacterial genera and their potentially active interaction were positively correlated to cellulose metabolism, some cellulose-metabolising degraders were represented poorly in pandas. Furthermore, some fungal genera that are the main degraders of complex cellulose in nature, such as *Aspergillus*, *Penicillium* and *Trichoderma,* were also found in pandas [26,27] particularly in cubs and adults. This indicates that fungi might also help their host to digest cellulose in food, whereas the numbers of these populations were also scant.

## 4. Discussion

### 4.1. Succession of Intestinal Microbial Structure and Its Potential Relationship with the Growth and Diet of Giant Pandas

In order to reasonably explore the succession regularity of intestinal microbes during their growth and development, different development stages in the long-life cycle of pandas (cub, sub-adult and adult) were investigated under similar growth environments. Twins were selected with similar physiological conditions and in the same development stage to maintain homogeneity. We found diversity and structurally characterized abundance of intestinal microbes in giant pandas at different developmental stages. Compared with other development stages, the intestinal bacterial flora structure of giant panda cubs was more complex, which may be related to the complex dietary shift process and active metabolism of giant panda cubs [14]. Therefore, it was speculated that the bacterial flora structure of giant panda cubs tends to be stable and that they initially have bamboo-eating adaptability, which was consistent with the results of the study of Guo et al. [15,19,20]. Then, from the sub-adult stage to the prime of life, the abundance and diversity of intestinal bacterial structure increased or at least remained stable, when giant pandas had formed their digestive physiology, indicating a specific succession of intestinal microbiota in pandas during the development and with increasing bamboo-adapting diet.

However, the complexity of intestinal bacterial structure of older adult giant pandas decreased [28]. Therefore, it was plausible to speculate that the maximum load may occur in the intestinal microecosystem during the most vigorous period of adult giant pandas’ lives [29]. This is the first time that the microbial diversity of giant panda with the formation of a specific bamboo diet has been analysed during almost the entire developmental stage. Obviously, when giant pandas start a restricted diet, both intestinal bacterial and fungal structure change accordingly. Especially before and after the sub-adult developmental stage, when pandas go through the dietary shift and increase bamboo intake, the microbial community structure changes greatly; the diversity of intestinal flora of adult pandas remained abundant and stable, explaining the adaptation to a restricted diet of the giant panda from the perspective of intestinal microbial homeostasis [30].

During the development of giant pandas, the change in bacterial flora abundance was contrary to that of fungi. Therefore, it was speculated that there is a certain competitive relationship between bacteria and fungi in the limited space of the micro-ecosystem in the intestines of giant pandas to keep the system steady [31,32]. The diversity and structure of fungi vary more greatly in the intestines of different older giant pandas, which may be influenced by environment, food and hygiene [33]. *Shiraia* mainly exists on bamboo, gradually and stably colonizing the gut of pandas with bamboo diets. Species *Aspergillus*, *Penicillium* and *Trichoderma* species can degrade lignocellulose in plants and have been found in the gut microbiome of our samples by sequencing [34]. In addition, genera such as Unclassified_o_Pleosporales, *Erythrobasidium* and *Trimmatostroma* are abundant in the faeces and expand with the development of pandas, and most of these fungi in the intestines are pathogenic of plant origin [35,36], but interestingly, the fungal flora structure in the intestines of giant pandas has always been relatively uniform. Martinez-Romero et al. have also found that the plant endophytes could form a stably and functionally microbial structure during long-term interaction with herbivorous animals [37,38]. There were no obviously predominant genera to colonize the intestines of giant pandas for a long time, which might be also a self-protection mechanism of the host for defending the conditional pathogenic microbiota. The higher dominance of intestinal fungi in older adult giant pandas may be due to the stable parasitic relationship formed by long-term interaction with intestinal fungi [32] or the decline of immunity [39]. Accordingly, the old adult pandas may have altered microbial diversity due to their diminished vigorous life period.

The dominant bacteria genera in the intestines of giant pandas at different developmental stages were mainly *Streptococcus*, *Escherichia-Shigella* and *Clostridium_sensu_stricto_1*, which is consistent with the research of Zhang et al. [15]. From the overall composition and structure of microbes, the intestine of the giant panda is still a typical carnivorous environment, which is also quite different from the microbial dominant species in herbivorous animals [12,40]. However, during the growth and development of giant panda, changes in the proportion of dominant bacteria in the intestines generally exist and are likely to be a result of the complex interaction between intestinal flora and its host, and long-term adaptation to the bamboo diet. For instance, the dominant *Streptococcus,* as was observed by Wang et al. [41], can degrade cellulose and was shown to be high in the intestines of the cubs. The relative abundance of *Streptococcus* increasing from 14.6% to 47.6%, especially after the cubs change feeding habits into a high-cellulose diet. *Clostridium* increased gradually during the giant pandas’ specialization of eating bamboo and especially occupied a dominant position in the intestine of sub adults’, who had just started to consume low-energy food [42]. The *Escherichia–Shigella* always maintained a high proportion of the intestinal bacterial community, and it was indeed correlated with many essential energy metabolism pathways in panda [19]. There was no obvious regularity in the structural changes of dominant fungi in the intestines, which was easily interfered [29,43]. While, it is possible to emphasize that most fungi are conditionally pathogenic, and the rapid increase in the number of fungi in the body to make it dominant will harm the health and development of its host [35].

On the whole, during the growth and development of giant pandas, the giant pandas in the development stage from sub-adult to adult carried the most stable intestinal flora structure, with no obvious difference between genders. In the actual process of feeding and managing giant pandas, human activities such as changing captivity, which may slightly affect the physiological health of giant pandas, should be scheduled at this stage as far as possible. However, the complexity of intestinal microbial structure of giant panda cubs was very high, even higher than that of adult pandas, which may be due to many influencing factors of intestinal flora of cubs, such as physiological environment in their maternal body, the external environments after birth and dietary shift process, which can change the structure of intestinal flora of cubs and even extend to the whole life [44]. The vulnerability of giant pandas was partly because of their poor breeding ability and low survival rate of cubs. Additionally, the imperfect and unstable intestinal flora structure of cubs may be one of the important reasons, especially at one year of age.

### 4.2. Intestinal Microbial Endocellulase Activity and Cellulose Digestibility of Giant Pandas at Different Developmental Stages

For a long time, giant panda cubs have adapted to bamboo in the stage of dietary shift [45]. In contrast with the findings of Zhang et al. [15], we found that when the giant panda cubs are in the process of the bamboo-consumption training process, their endocellulase (a key enzyme for cellulose degradation) has already been already active in gut, especially when they are 1–1.5 years old. However, the intestinal endocellulase activity of the sub-adult giant panda decreased significantly and then increased in adults. It is an unexpected result that the cubs without a long-term bamboo diet have higher endocellulase activity than those in any other stage. We speculate that there may be two main reasons. Firstly, the cubs have a stress response to the high content of bamboo cellulose by increasing cellulase activity, and secondly the endocellulase of cubs is isoenzymes, but they do not act on bamboo cellulose. However, further studies are warranted to prove these hypotheses.

Considering changes in endocellulase activity in pandas, the dominant *Streptococcus* processed a similar fluctuation to it. However, both the abundance of *Streptococcus* and the cellulase activity decreased in cubs when they began to consume a bamboo diet, which suggests that *Streptococcus* was likely an important genus to metabolize cellulose in supplementary food but not in bamboo in cubs. Although *Clostridium* also kept a certain amount and were reported to have the ability to assist cellulose degradation [15,43], unfortunately, it contributed less to cellulase activity. Therefore, not all the potential cellulolytic species in the observed bacterial genus have the ability to metabolize cellulose in pandas. To clarify whether the succession of microbial dominant species in the gut is linked with the digestion of refractory polysaccharides in bamboo during the development of pandas under the specific digestive physiology, we need to further analyse the degrading ability to specific polysaccharides of a certain species, or flora, based on culturing in vitro [46]. In fact, some cellulose degraders such as *Pseudomonas*, *Paenibacillus* and *Sphingobacterium* among bacteria and *Aspergillus*, *Penicillium* and *Trichoderma* among fungi were represented with low abundance in pandas. In general, according to the cellulase activity in pandas, the digestibility of gut microbes to bamboo cellulose was still poor, though the adults increased their enzymic activity with bamboo diet.

Studies have found that in the process of adapting to bamboo eating, giant pandas not only accumulated dominant flora which had potential for cellulose degradation but also maximized the use of nutrients such as protein, amino acid and fatty acid in food through other flora or evolved metabolism [47,48]. It was speculated that sub-adult giant pandas can adjust their digestion and absorption and form a set of nutrition absorption mode suitable for their growth and development, which may include changes in intestinal microbial structure. The intestinal microbial structure of sub-adult giant panda may determine its ability in the later vigorous growth stage to digest and absorb bamboo cellulose, protein and other components. Further, through correlation analysis and pathway analysis, we have a deeper understanding of the influence of the interaction between intestinal microbes on the cellulose digestion ability of giant pandas [49]. For instance, the abundance of *Streptococcus* increased, and it was shown that a positive correlation with the cellulase activity in adult pandas with bamboo diet depended on the interaction of other microflora, although it also preferred to use oligosaccharides such as *Escheriachia-Shigella* [20] and dietary fibre in supplementary food. Therefore, these findings reveal the important influence of the social behaviour of intestinal microbes on the growth and nutrition metabolism of giant pandas. However, studying more complex interactions of intestinal microbes relies on a large individual base. This research is still inadequate but provides some preliminary findings of correlations between dominant flora. These findings advance our understanding of the evolution of giant pandas adapting to bamboo, and by increasing the individual numbers and improving analysis methods, it is expected that more vivid co-evolutionary knowledge will be obtained about the gut microbes and pandas with bamboo diet.

## 5. Conclusions

Taken together, the intestinal microbial structure of giant pandas varied at different developmental stages. The diversity of intestinal bacteria was high in the cub stage, and the structure of bacterial flora tends to be stable after the consumption bamboo diet during adaptation to eating bamboo. In the adult development stage, the complexity of the intestinal bacterial structure of giant pandas may be at its highest point, which was also the most vigorous period of giant pandas. The flora abundance of fungi showed an opposite trend to that of bacteria. In the limited space of the micro-ecosystem in the intestines of giant pandas, there was a certain strict relationship between bacteria and fungi, which maintained the system steady. The dominant bacterial genera in the intestines of giant pandas showed obvious dominance and at different developmental stages were mainly *Streptococcus, Escherichia-Shigella* and *Clostridium_sensu_stricto_1*. The structure of intestinal dominant fungi carried no obvious regularity with age. During the growth of giant pandas, the activity of intestinal cellulase was obviously different in different periods with the order of cubs > adults > sub-adults. The sub-adult stage was the turning point, and its intestinal microbial structure may determine its ability to digest and absorb bamboo cellulose, protein and other components in the later growth and development stage when they have vigorous metabolism. However. bamboo cellulose is still not a useful substrate for the dominant intestinal microbes of giant panda to obtain energy and nutrition after eating a bamboo diet.

## Figures and Tables

**Table 1:** Results of basic information of experimental giant panda.

Name	Lineage	Date of Birth	Sex	Weight (m/kg)	Location	Health Condition	SampleNumber
Yueyue	1052	4 October 2016	Male	33.6	Shanghai wild animal park	Health	S1
Banban	1053	4 October 2016	Female	32.4	Shanghai wild animal park	Health	S2
Xinger	900	23 August 2013	Male	112.3	Shanghai zoo	Health	S3
Yaer	904	27 August 2013	Male	116.8	Shanghai zoo	Health	S4
Yaao	583	13 August 2004	Male	136.0	Shanghai wild animal park	Health	S5
Youyou	474	14 July 1998	Female	108.0	Shanghai wild animal park	Health	S6

**Table 2:** Intestine cellulase activity of giant panda at different developmental stages.

Sample	Aug (2017)	Feb (2018)	Average
S1	12.02 ± 1.518	31.43 ± 1.037	21.72 ± 13.722
S2	15.08 ± 0.815	34.67 ± 0.444	24.89 ± 13.851
S3	2.32 ± 0.130	8.07 ± 1.037	5.19 ± 4.064
S4	0.45 ± 0.037	2.72 ± 0.296	1.58 ± 1.612
S5	2.07 ± 0.667	14.98 ±1.037	8.52 ± 9.129
S6	13.61 ± 0.500	23.36 ± 0.296	18.48 ± 6.898

**Table 3:** Path analysis of cellulase activity (y) in giant panda intestine and relative abundance (Xi) of intestinal dominant flora.

Variable (x_i_)	Simple Correlation Coefficient with y (r_iy_)	Direct Path Coefficient (P_iy_)	Indirect Path Coefficient (x_j_)				Total ∑(r_ij_ × P_jy_)
			X_1_ (r_i1_ × P_1y_)	X_2_ (r_i2_ × P_2y_)	X_3_ (r_i3_ × P_3y_)	X_4_ (r_i4_ × P_4y_)	
X_1_	−0.954	−1.112	-	0.563	−0.753	0.348	0.158
X_2_	0.857	−0.655	0.955	-	0.889	−0.333	1.511
X_3_	−0.852	−1.056	−0.792	0.552	-	0.444	0.203
X_4_	−0.835	0.484	−0.798	0.450	−0.969	-	−1.317

## Data Availability

The datasets generated in this study can be found in the NCBI, PRJNA751754 and Supplementary files.

## Supplementary Materials

The following are available online at https://www.mdpi.com/article/10.3390/ani11082358/s1, Table S1: Intestinal bacterial diversity index of giant pandas at different developmental stages, Table S2:. Intestinal fungal alpha diversity index of giant pandas at different developmental stages, Table S3: Relative abundance of three dominant bacteria in the gut of giant pandas at different developmental stages, Table S4: Pearson correlation analysis of cellulase activity and relative abundance of intestinal dominant flora in Giant Panda at different development stages.
