# Supplementary material for: Succession of Intestinal Microbial Structure of Giant Pandas (Ailuropoda melanoleuca) during Different Developmental Stages and Its Correlation with Cellulase Activity
Source: Animals (Basel). 2021 Aug 10;11(8):2358. doi: 10.3390/ani11082358 (PMC8388744; doi:10.3390/ani11082358)
Supplement: Supplementary file 1 [file animals-11-02358-s001.zip › animals-1188372-supplementary.pdf]

Table S1. Intestinal bacterial diversity index of giant pandas at different developmental stages

Sample	Sobs		Shannon index		Simpson index		Chao		Coverage	
	Aug (2017)	Feb (2018)	Aug (2017)	Feb (2018)	Aug (2017)	Feb (2018)	Aug (2017)	Feb (2018)	Aug (2017)	Feb (2018)
S1	93	188	1.95	1.86	0.24	0.27	112.13	256.55	0.999	0.999
S2	113	163	2.55	1.77	0.13	0.27	138.67	218.57	0.999	0.999
S3	89	83	1.39	1.68	0.41	0.26	114.3	153.13	0.999	0.999
S4	100	54	1.36	1.70	0.44	0.24	133.83	69.6	0.999	0.999
S5	94	152	1.48	1.67	0.32	0.26	131.8	294.06	0.999	0.998
S6	94	98	1.27	0.98	0.39	0.52	130.25	167.46	0.999	0.999

Table S2. Intestinal fungal alpha diversity index of giant pandas at different developmental stages.

Sample	Sobs		Shannon index		Simpson index		Chao		Coverage	
	Aug (2017)	Feb (2018)	Aug (2017)	Feb (2018)	Aug (2017)	Feb (2018)	Aug (2017)	Feb (2018)	Aug (2017)	Feb (2018)
S1	118	406	4.01	2.92	0.03	0.10	123.60	525.25	0.999	0.998
S2	145	397	2.64	3.22	0.13	0.10	232.88	437.30	0.999	0.999
S3	443	385	3.67	3.40	0.07	0.07	466.43	420.47	0.999	0.999
S4	335	335	3.17	3.17	0.09	0.09	351.53	351.53	0.999	0.999
S5	216	427	3.43	3.40	0.08	0.08	222.50	482.94	0.999	0.998
S6	232	365	2.53	3.15	0.29	0.16	242.50	410.00	0.999	0.999

Table S3. Relative abundance of three dominant bacteria in the gut of giant pandas at different developmental stages

Sample	*Streptococcus*		*Escherichia-Shigella*		*Clostridium*	
	Aug(2017)	Feb(2018)	Aug(2017)	Feb(2018)	Aug(2017)	Feb(2018)
S1	14.5%	49.2%	32.8%	17.5%	5%	14.5%
S2	14.7%	46%	26.8%	25.7%	11.3%	12.8%
S3	4%	40%	58.5%	14.9%	26.5%	35.3%
S4	3%	40%	63.1%	22.2%	22%	29%
S5	33.1%	33.5%	45.5%	24.7%	14.5%	32.9%
S6	50%	70%	39.4%	26%	8%	1%

Table S4. Pearson correlation analysis of cellulase activity and relative abundance of intestinal dominant flora in Giant Panda at different development stages

	Enzyme	*Streptococcus*	*Escherichia-Shigella*	*Clostridium-_sensu_stricto_1*	*Turicibacter*	*Lactobacillus*	Unclassified_f_Montagnulaceae	*Trichosporon*	*Trimmatostroma*	Unclassified_f_norank_o_Pleosporale	Unclassified_f_Apiosporaceae	Unclassified_o_Pleosporales	*Leptoxyphium*	*Shiraia*
Enzyme	1	.857*	-.954**	-.835*	.623	.806	-.166	.684	-.502	-.104	-.852*	.364	.293	-.376
	6	6	6	6	6	6	6	6	6	6	6	6	6	6
Streptococcus		1	-.859*	-.687	.768	.760	-.140	.460	-.508	.295	-.842*	.157	.093	.049
		6	6	6	6	6	6	6	6	6	6	6	6	6
Escherichia-Shigella			1	.718	-.745	-.860*	.052	-.547	.301	.102	.713	-.205	-.145	.416
			6	6	6	6	6	6	6	6	6	6	6	6
Clostridium-_sensu_stricto_1				1	-.204	-.428	.509	-.385	.612	.362	.918**	-.764	-.717	.309
				6	6	6	6	6	6	6	6	6	6	6
Turicibacter					1	.921**	.488	.497	.073	.369	-.393	-.454	-.507	-.145
					6	6	6	6	6	6	6	6	6	6
Lactobacillus						1	.429	.726	-.019	.097	-.544	-.229	-.297	-.429
						6	6	6	6	6	6	6	6	6
Unclassified_f_Montagnulaceae							1	.285	.748	.159	.382	-.859*	-.868*	-.306
							6	6	6	6	6	6	6	6
Trichosporon								1	-.321	.102	-.532	-.041	-.124	-.356
								6	6	6	6	6	6	6
Trimmatostroma									1	-.253	.713	-.642	-.593	-.394
									6	6	6	6	6	6
Unclassified_f_norank_o_Pleosporale										1	-.010	-.532	-.558	.803
										6	6	6	6	6
Unclassified_f_Apiosporaceae											1	-.556	-.492	.054
											6	6	6	6
Unclassified_o_Pleosporales												1	.996**	-.131
												6	6	6
Leptoxyphium													1	-.120
													6	6
Shiraia														1
														6

* $P < 0.05$; ** $P < 0.01$.
